# Supplementary material for: Historic mine waste contains diverse microbial communities that reflect waste type and geochemistry
Source: Appl Environ Microbiol. 2025 Jul 24;91(8):e00434-25. doi: 10.1128/aem.00434-25 (PMC12366339; doi:10.1128/aem.00434-25)
Supplement: Supplemental figures — Figures S1 to S9. [file aem.00434-25-s0001.pdf]

Supplementary Figures and Tables

Historic mine waste contains diverse microbial communities that reflect waste type and geochemistry

Mackenzie B. Best[1], Zohreh Kazemi Motlagh[2], Virginia T. McLemore[3], and Daniel S. Jones[1,4]

[1]Department of Earth and Environmental Science, New Mexico Institute of Mining and Technology, Socorro, New Mexico, USA
[2]Department of Mineral Engineering, New Mexico Institute of Mining and Technology, Socorro, New Mexico, USA
[3]New Mexico Bureau of Geology and Mineral Resources, New Mexico Institute of Mining and Technology, Socorro, New Mexico, USA
[4]National Cave and Karst Research Institute, Carlsbad, New Mexico, USA

SUPPLEMENTARY TABLE S1 Summary and associated metadata for all samples.
(Uploaded separately)

SUPPLEMENTARY TABLE S2 Cell counts from select waste rock and tailings

Sample	Location	Sample type	Cells/g wet sediment	Sample notes
**AH24-13**	Alhambra	bulk waste	9.9×10^{8}	soil cover, waste composite
**BH24-13**	Black Hawk	bulk waste	1.6×10^{9}	soil cover, waste composite
**BH24-23**	Black Hawk	bulk waste	1.5×10^{9}	soil cover, waste composite
**CN23-13**	Center	bulk waste	1.1×10^{10}	soil cover, waste composite
**CY23-13**	Carlisle	bulk waste	3.9×10^{9}	waste composite
**CF22-601***	Copper Flat	bulk waste	2.4×10^{8}	
**CF22-603***	Copper Flat	bulk waste	1.2×10^{8}	
**CF22-604***	Copper Flat	bulk waste	6.1×10^{7}	
**CF22-606***	Copper Flat	bulk waste	4.7×10^{8}	
**CF23-621**	Copper Flat	tailings	1.0×10^{8}	above oxidation front
**CF23-622**	Copper Flat	tailings	9.0×10^{7}	at oxidation front
**CF23-623**	Copper Flat	tailings	2.0×10^{7}	below oxidation front
**CF23-631**	Copper Flat	tailings	4.8×10^{7}	above oxidation front, tailings composite
**CY23-20**	Carlisle	tailings	9.7×10^{7}	above oxidation front
**CY23-21**	Carlisle	tailings	6.9×10^{8}	at oxidation front
**CY23-22**	Carlisle	tailings	1.6×10^{8}	below oxidation front

*Individual samples (composite sample was not available for cell counting from the CF composite)

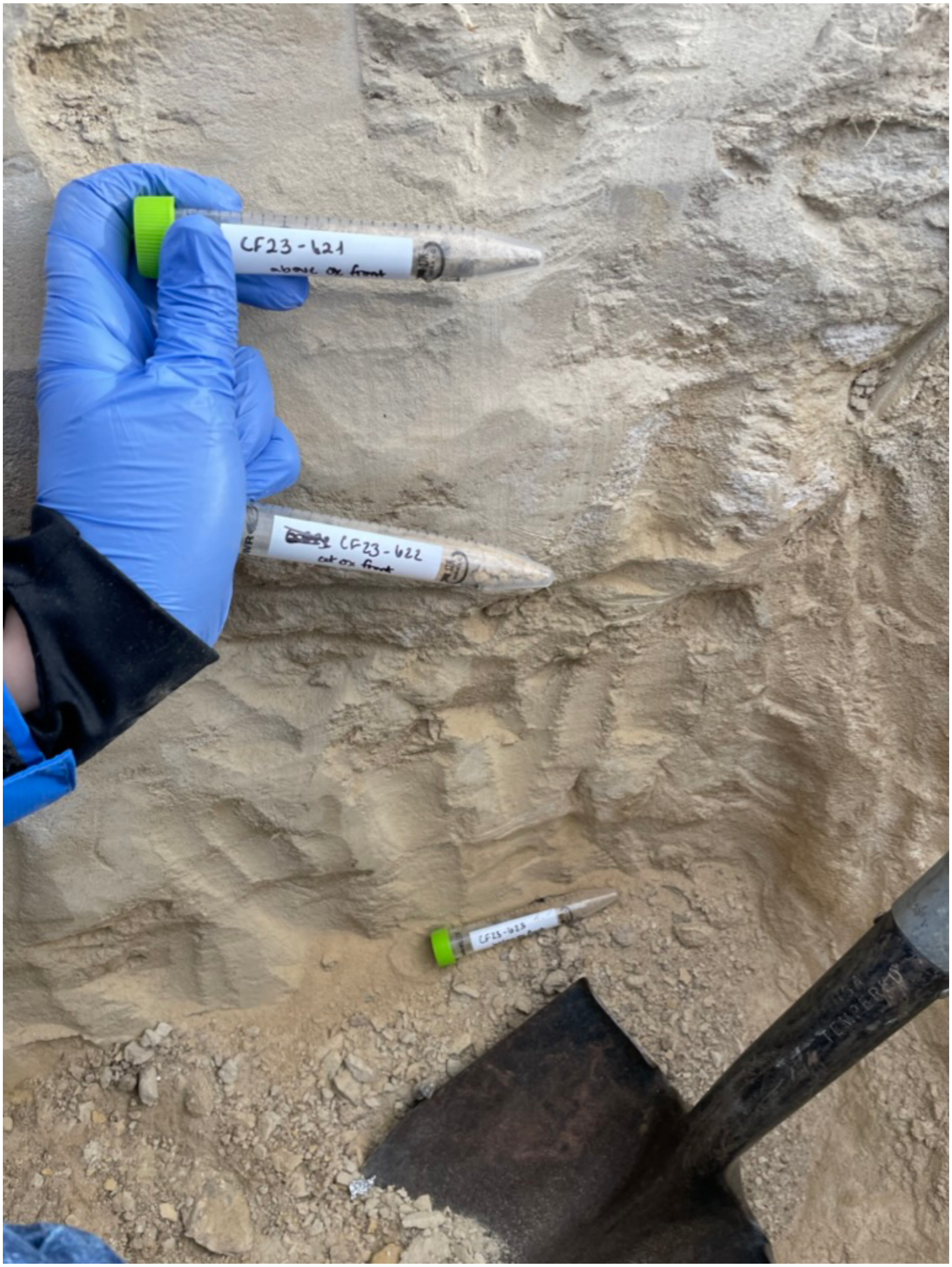

SUPPLEMENTARY FIG S1. Tailings samples collected from a 1 m pit at Copper Flat Mine. Samples were collected from above, at, and below the oxidation front, which is visible due to color changes from white, to beige, to yellow.

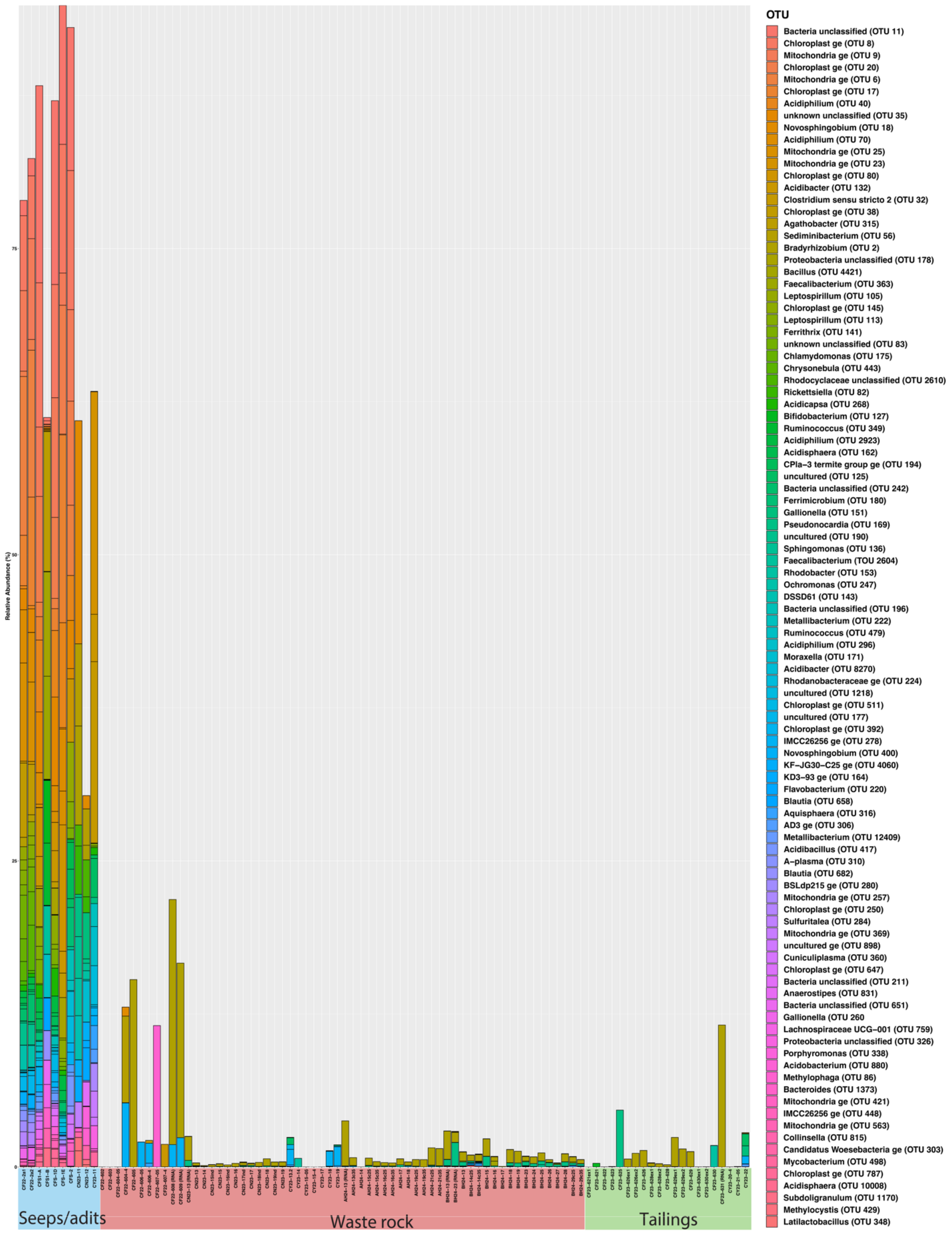

SUPPLEMENTARY FIG S2. Genus-level microbial community composition of the 100 most abundant OTUs in the acidic seep and adit samples. Bars representing samples are grouped by sample type (L to R): seeps/adits, bulk waste rock, and tailings.

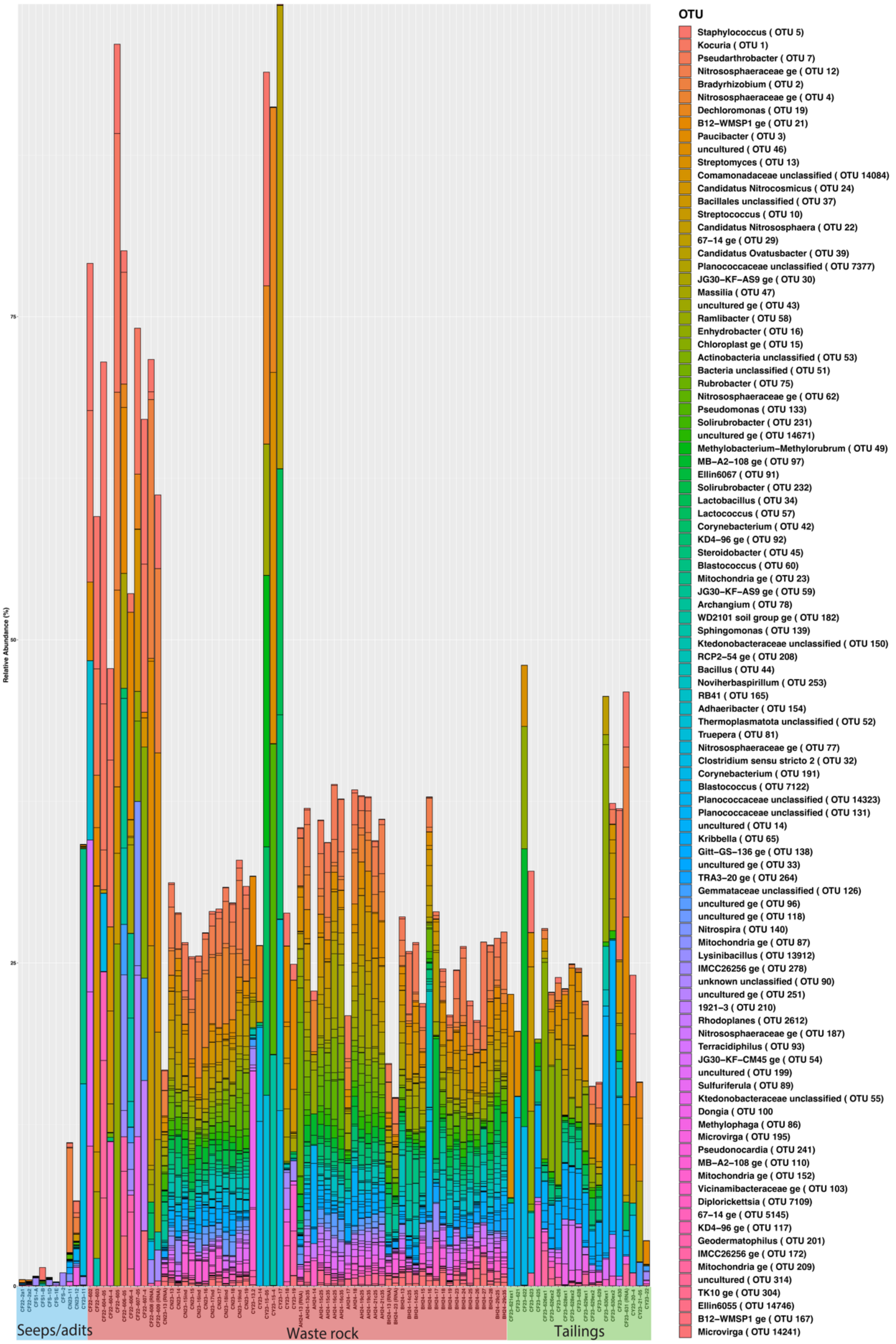

SUPPLEMENTARY FIG S3. Genus-level microbial community composition of the 100 most abundant OTUs in the bulk waste rock samples. Bars representing samples are grouped by sample type (L to R): seeps/adits, bulk waste rock, and tailings.

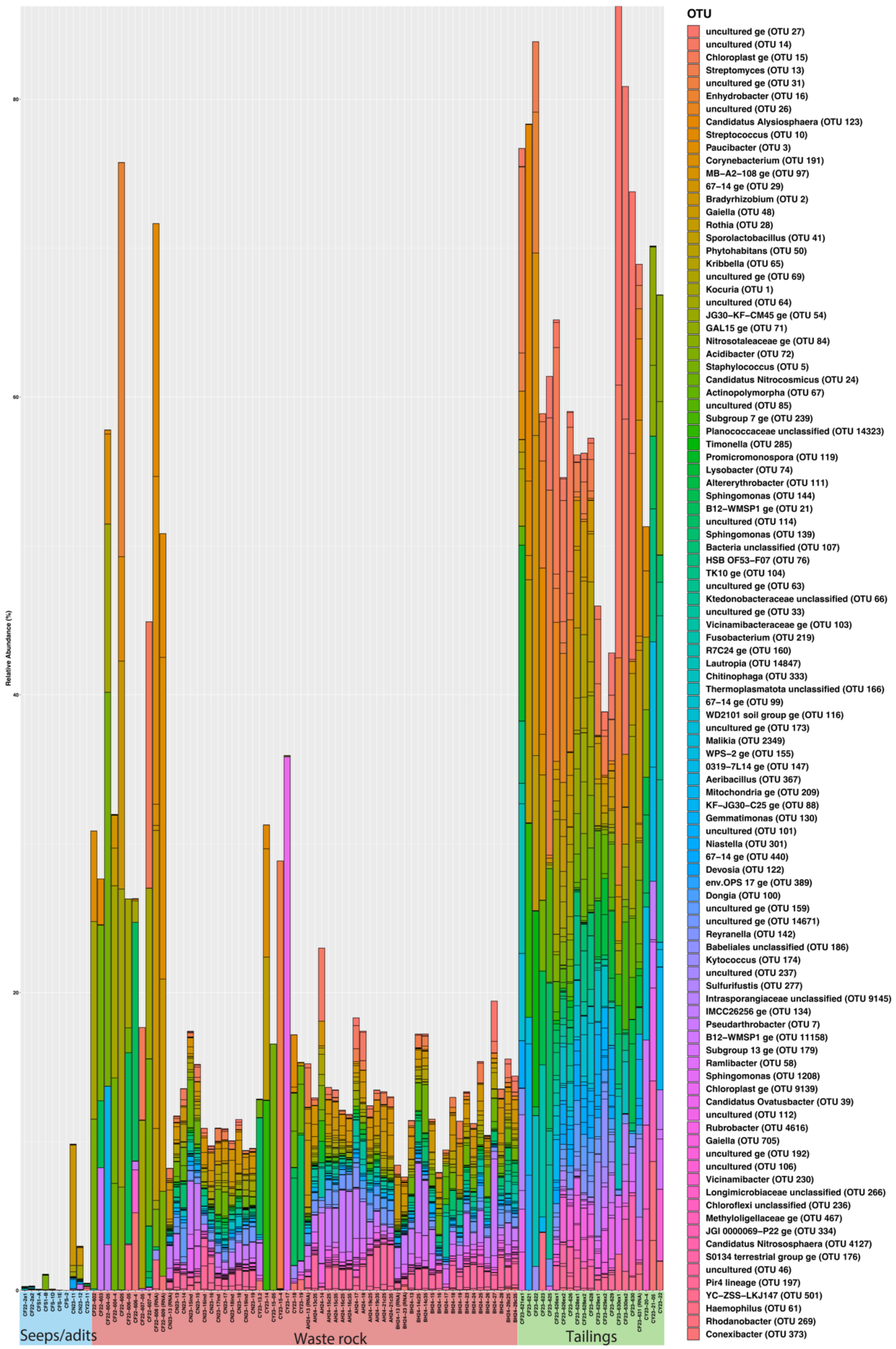

SUPPLEMENTARY FIG S4. Genus-level microbial community composition of the 100 most abundant OTUs in the tailings samples. Bars representing samples are grouped by sample type (L to R): seeps/adits, bulk waste rock, and tailings.

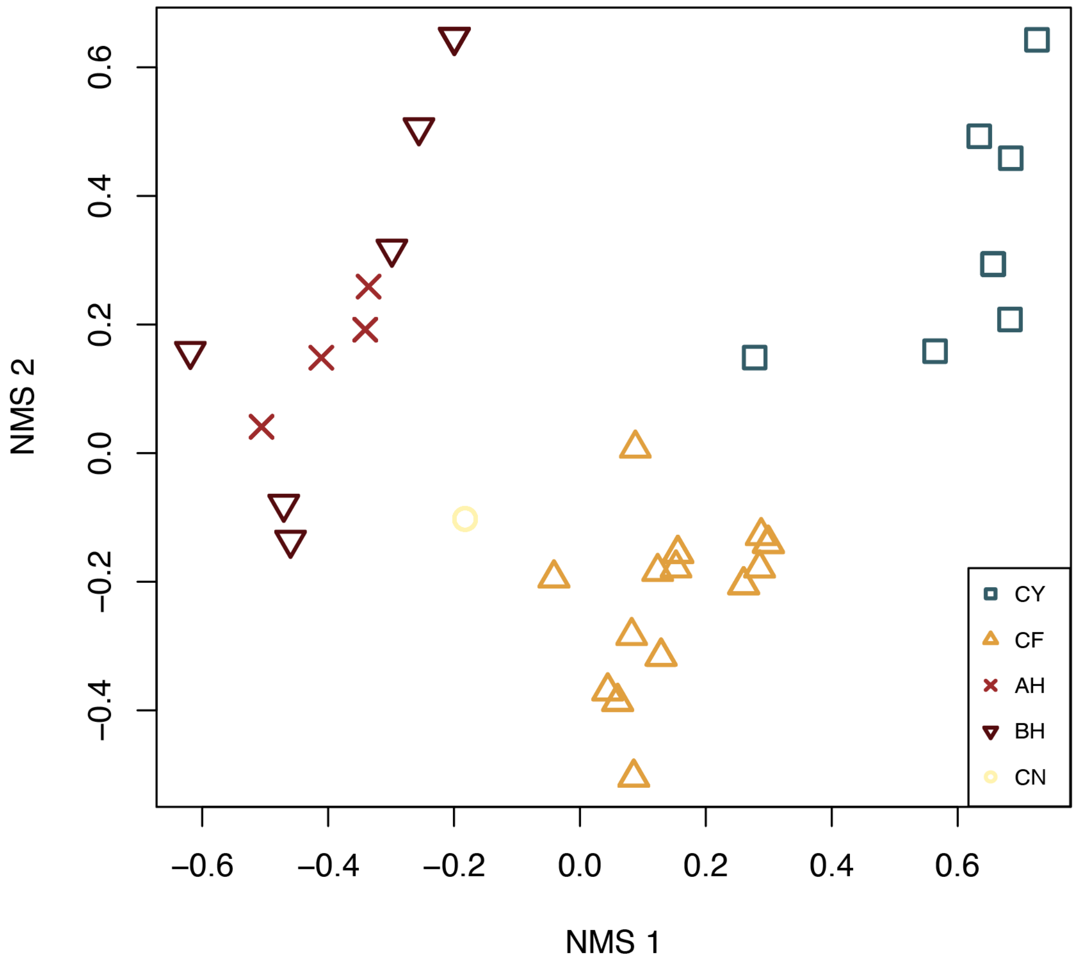

SUPPLEMENTARY FIG S5. Non-metric multidimensional scaling (NMDS) ordination of individual mine sites based on geochemical parameters alone. Parameters used to ordinate samples are carbon (%), sulfur (%), gold (ppm), silver (ppm), arsenic (ppm), cobalt (ppm), copper (ppm), iron (%), lead (ppm), total rare earth elements (TREE; ppm), uranium (ppm), and silica (%) (Supplementary Table S1). CY = Carlisle Mine, CF = Copper Flat Mine, AH = Alhambra Mine (AH), BH = Black Hawk Mine, Center Mine = CN.

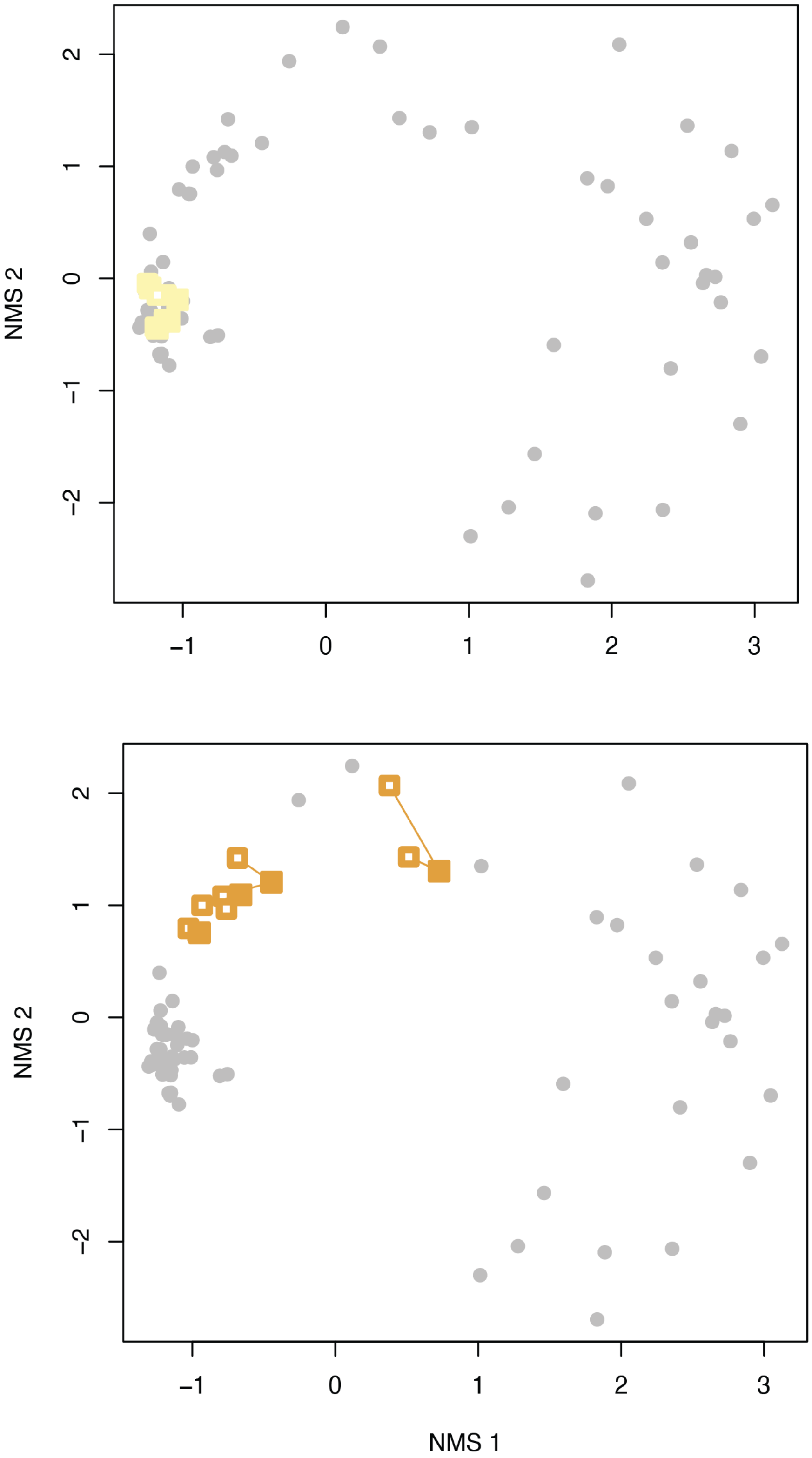

SUPPLEMENTARY FIG S6. A non-metric multidimensional scale (NMDS) ordination of samples from Center Mine (top, yellow) and Copper Flat Mine (bottom, orange) showing the similarity between individual DNA extracts (open squares) and the combined DNA extract (closed squares).

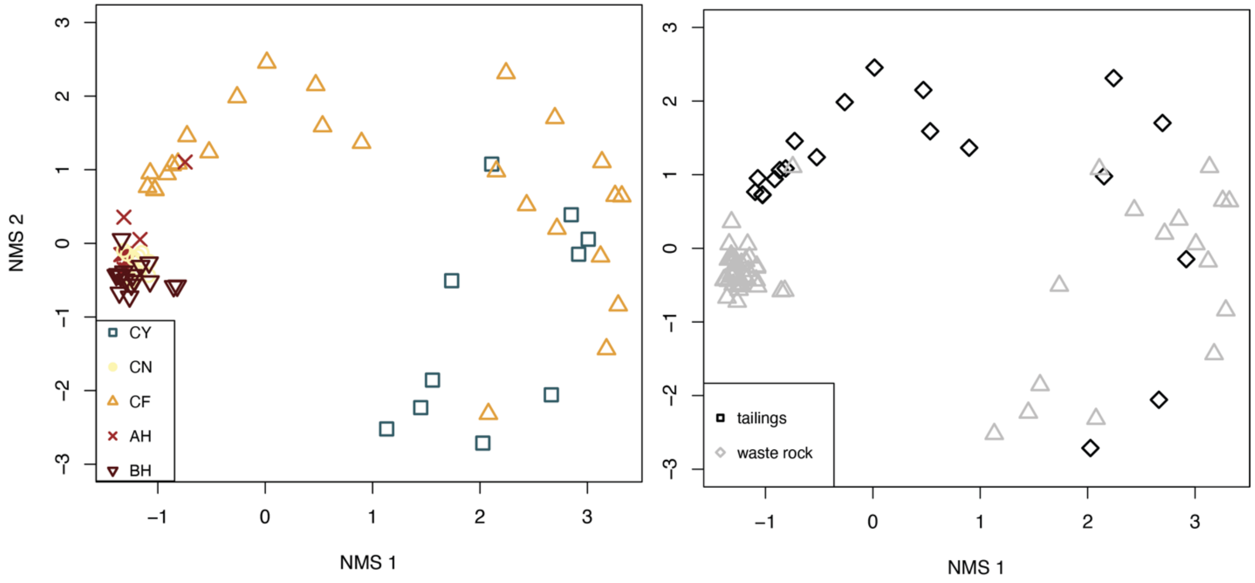

SUPPLEMENTARY FIG S7. Non-metric multidimensional scaling (NMDS) ordination of rRNA gene libraries only (no rRNA transcript libraries like in Figure 4 in the main text). Left: Samples are coded by location with samples from Center Mine (CN) as open yellow circles, Copper Flat Mine (CF) samples represented by open orange triangles, Alhambra Mine (AH) samples as red x's, Black Hawk Mine (BH) as dark red open upside-down triangles, and Carlisle Mine (CY) as open blue squares. Right: Samples are coded by sample type where tailings samples are represented by open black squares and waste rock samples are shown with open grey triangles.

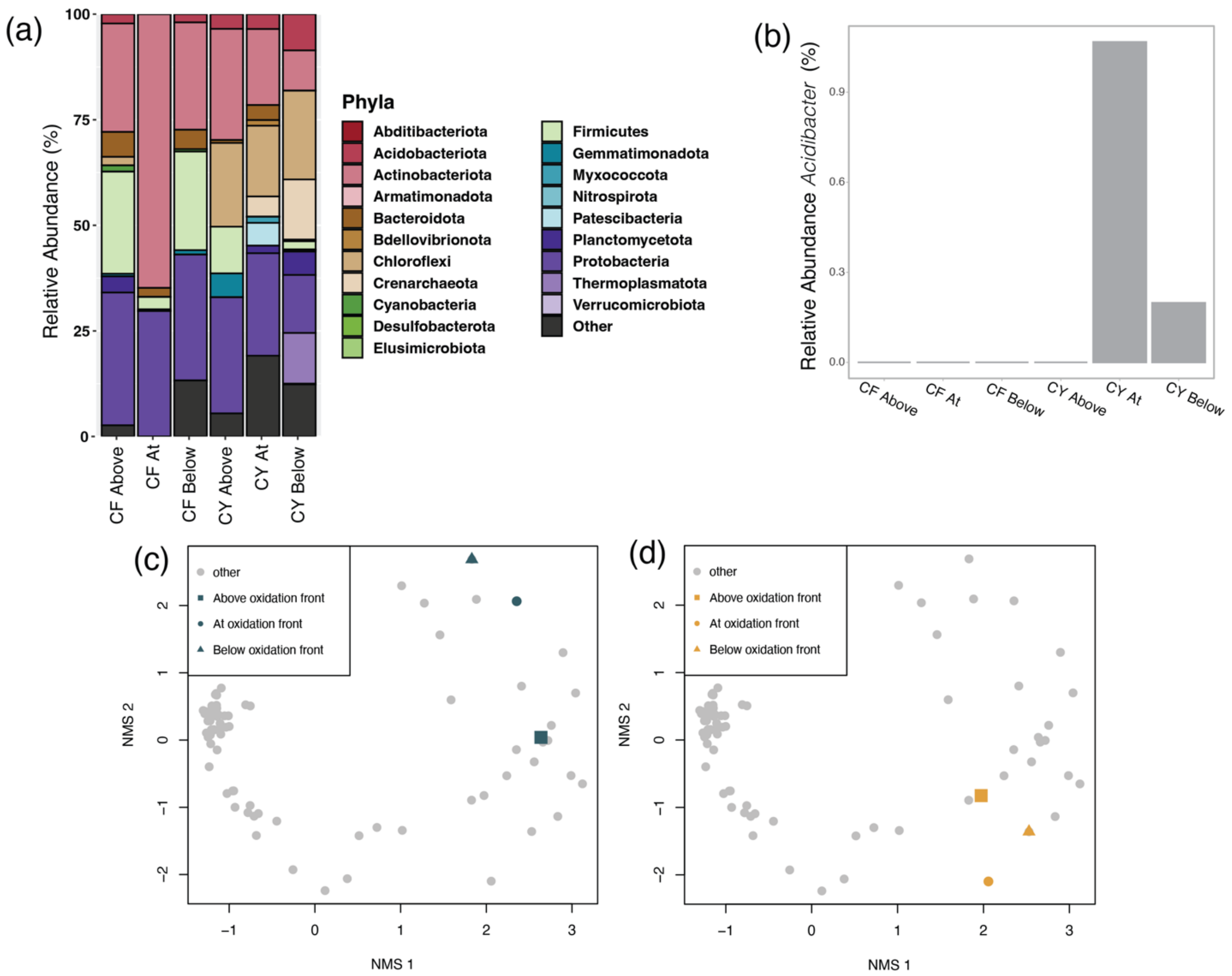

SUPPLEMENTARY FIG S8. (a) Phyla-level community composition of samples collected from Copper Flat (CF) and Carlisle Mines (CY) above, at, and below the visible redox interface (Fig. S1, S2). (b) The relative abundance of *Acidibacter* spp. across the redox interface at CF and CY. (c) NMDS ordination comparing microbial communities across the redox interface at CY. (d) NMDS ordination comparing microbial community composition across the redox interface at CF.

SUPPLEMENTARY FIG S9 (next two page). Correlations between select OTUs (operational taxonomic units) and geochemical parameters. The text at the top of the figure indicates Pearson's correlation coefficient and statistical significance (R and p-value) for the relationship shown. The taxonomic affiliation for the OTUs includes phylum- and genus-level classifications, or the highest available taxonomic classification for uncultured taxa. Confidence scores >50 are provided in parentheses.

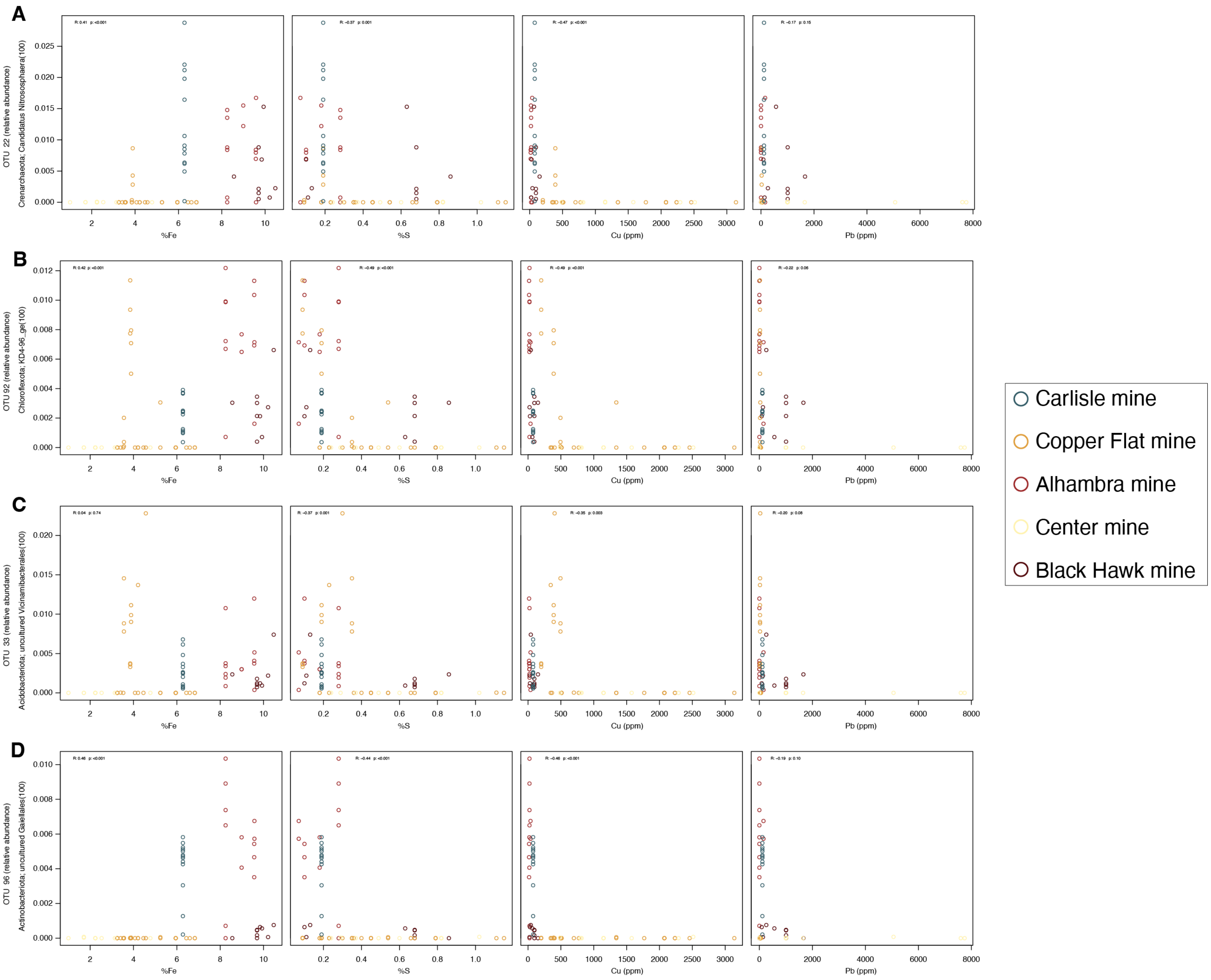
A
OTU 22 (relative abundance)
Crenarchaeota; Candidatus Nitrososphaera(100)
R: 0.41 p: <0.001
R: −0.37 p: 0.001
R: −0.47 p: <0.001
R: −0.17 p: 0.15
%Fe
%S
Cu (ppm)
Pb (ppm)
B
OTU 92 (relative abundance)
Chloroflexota; KD4-96_ge(100)
R: 0.42 p: <0.001
R: −0.49 p: <0.001
R: −0.49 p: <0.001
R: −0.22 p: 0.06
%Fe
%S
Cu (ppm)
Pb (ppm)
C
OTU 33 (relative abundance)
Acidobacteriota; uncultured Vicinamibacterales(100)
R: 0.04 p: 0.74
R: −0.37 p: 0.001
R: −0.35 p: 0.003
R: −0.20 p: 0.08
%Fe
%S
Cu (ppm)
Pb (ppm)
D
OTU 96 (relative abundance)
Actinobacteriota; uncultured Gaiellales(100)
R: 0.46 p: <0.001
R: −0.44 p: <0.001
R: −0.46 p: <0.001
R: −0.19 p: 0.10
%Fe
%S
Cu (ppm)
Pb (ppm)
Carlisle mine
Copper Flat mine
Alhambra mine
Center mine
Black Hawk mine

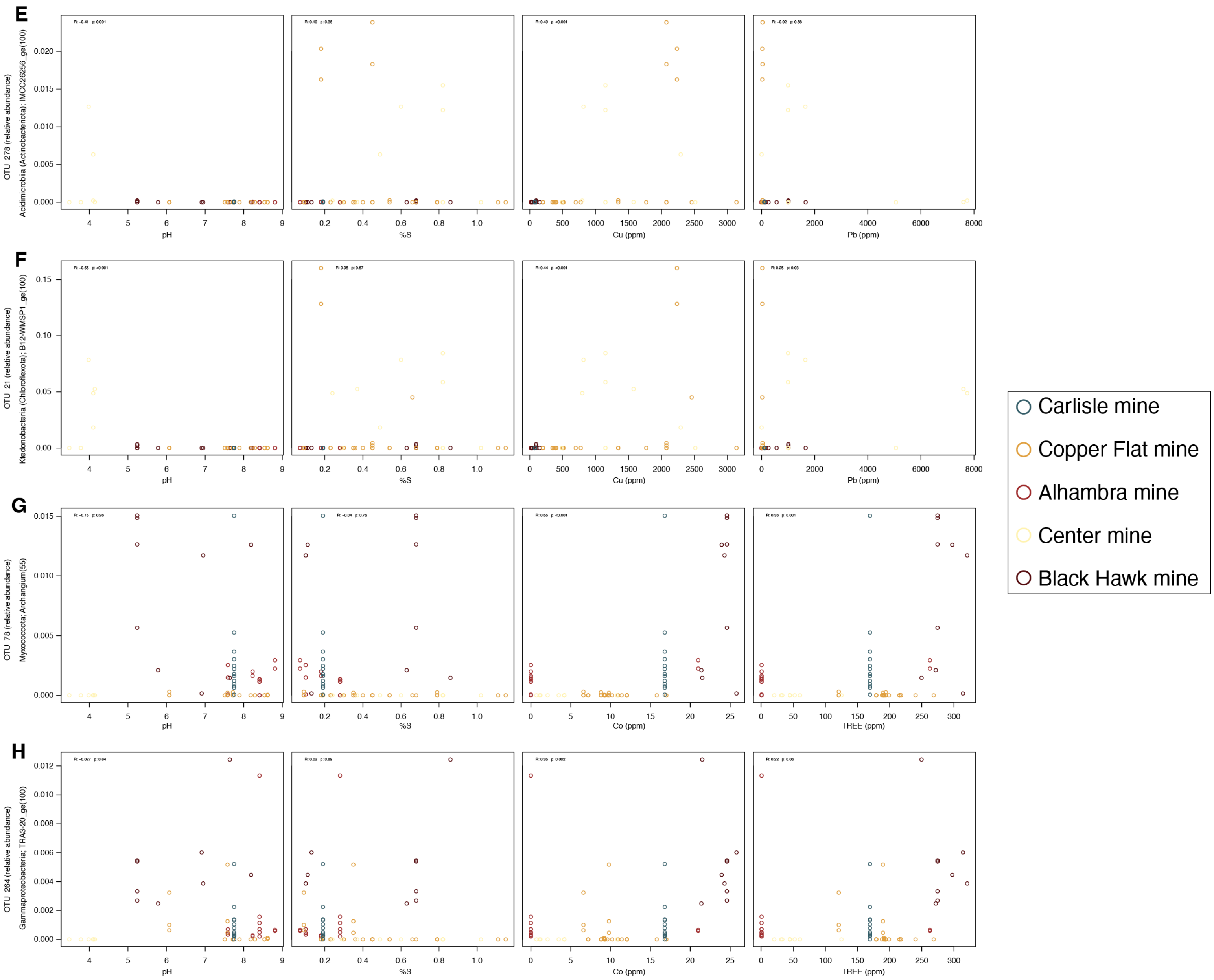
E
F
G
H
OTU_278 (relative abundance)
Acidimicrobiia (Actinobacteriota); IMCC26256_ge(100)
OTU_21 (relative abundance)
Ktedonobacteria (Chloroflexota); B12-WMSP1_ge(100)
OTU_78 (relative abundance)
Myxococcota; Archangium(55)
OTU_264 (relative abundance)
Gammaproteobacteria; TRA3-20_ge(100)
pH
%S
Cu (ppm)
Pb (ppm)
Co (ppm)
TREE (ppm)
Carlisle mine
Copper Flat mine
Alhambra mine
Center mine
Black Hawk mine
